# Visualization of the medial forebrain bundle using diffusion tensor imaging

**DOI:** 10.3389/fnana.2015.00139

**Published:** 2015-10-31

**Authors:** Ardian Hana, Anisa Hana, Georges Dooms, Hans Boecher-Schwarz, Frank Hertel

**Affiliations:** ^1^National Service of Neurosurgery, Centre Hospitalier de LuxembourgLuxembourg City, Luxembourg; ^2^Internal Medicine, Erasmus University of RotterdamRotterdam, Netherlands; ^3^Service of Neuroradiology, Centre Hospitalier de LuxembourgLuxembourg City, Luxembourg

**Keywords:** white matter tracts, medial forebrain bundle, diffusion tensor imaging, deep brain stimulation, cerebral lesions, neurosurgery

## Abstract

Diffusion tensor imaging is a technique that enables physicians the portrayal of white matter tracts *in vivo*. We used this technique in order to depict the medial forebrain bundle (MFB) in 15 consecutive patients between 2012 and 2015. Men and women of all ages were included. There were six women and nine men. The mean age was 58.6 years (39–77). Nine patients were candidates for an eventual deep brain stimulation. Eight of them suffered from Parkinson‘s disease and one had multiple sclerosis. The remaining six patients suffered from different lesions which were situated in the frontal lobe. These were 2 metastasis, 2 meningiomas, 1 cerebral bleeding, and 1 glioblastoma. We used a 3DT1-sequence for the navigation. Furthermore T2- and DTI- sequences were performed. The FOV was 200 × 200 mm^2^, slice thickness 2 mm, and an acquisition matrix of 96 × 96 yielding nearly isotropic voxels of 2 × 2 × 2 mm. 3-Tesla-MRI was carried out strictly axial using 32 gradient directions and one b0-image. We used Echo-Planar-Imaging (EPI) and ASSET parallel imaging with an acceleration factor of 2. *b*-value was 800 s/mm^2^. The maximal angle was 50°. Additional scanning time was < 9 min. We were able to visualize the MFB in 12 of our patients bilaterally and in the remaining three patients we depicted the MFB on one side. It was the contralateral side of the lesion. These were 2 meningiomas and one metastasis. Portrayal of the MFB is possible for everyday routine for neurosurgical interventions. As part of the reward circuitry it might be of substantial importance for neurosurgeons during deep brain stimulation in patients with psychiatric disorders. Surgery in this part of the brain should always take the preservation of this white matter tract into account.

## Introduction

Diffusion tensor imaging (DTI) is a technique that allows physicians the portrayal of white matter tracts (WMT) *in vivo* in healthy and non-healthy patients in a non-invasive way (Mesulam, 2005; Stadlbauer et al., 2006; Ciccarelli et al., 2008). It uses the differences in water molecule diffusion patterns along axon bundles throughout the brain (Basser et al., 1994; Ciccarelli et al., 2008). Using this technique we get important informations about the magnitude of diffusion anisotropy and the orientation of the maximum diffusion (Ciccarelli et al., 2008). Application areas include stroke, epilepsy, neurosurgical interventions, spinal cord disorders, or neurodegenerative disorders like Parkinson‘s disease (PD) (Ciccarelli et al., 2008). Some of the WMTs which are portrayed by means of DTI include the corticospinal tract (CST), visual pathway (VP), or the medial forebrain bundle (MFB). The MFB seems to be a structure of loosely attached fibers that connects important brain areas together, among others the ventral tegmental area (VTA) of the midbrain, the lateral hypothalamus and the septal area (Coenen et al., 2009a). This WMT is composed of ascending and descending tributaries which differ in their length (Coenen et al., 2009a). Studies suggest that this WMT is situated medially of the subthalamic nucleus (STN) (Coenen et al., 2009a). This would explain why electrodes which are put in the brain for deep brain stimulation (DBS) in patients with PD provoke side effects when they lie medially of the STN (Coenen et al., 2009a). However, the concrete localization of the MFB in humans seems to be disputed between experts. Some physicians depict it as a connection between upper brain stem and the hypothalamus whereas others see it as a WMT which starts anteriorly of the STN (Coenen et al., 2009a). Physicians consider the MFB to be an important part of the mesolimbic dopamine reward system. It is considered to be the neural substrate for the reward circuitry (Gálvez et al., 2015). There have been studies in rodents concerning this WMT too. They describe it as a steak-like structure which is mainly located in the lateral wall of the hypothalamus (Coenen et al., 2009a). There it represents a compact heterogeneous pathway (Coenen et al., 2011). It starts from the VTA and ends in different forebrain structures containing myelinated and unmyelinated fibers (Furlanetti et al., 2015). In contrast to this finding, the MFB seems to be more curved in the humans. In humans it is a bipartite structure with a common trunk at the beginning which splits into two pieces in the VTA of the midbrain (Coenen et al., 2012). Before that, it connects cerebellar nuclei with the VTA (Coenen et al., 2009a, 2011, 2012). In humans we have the inferomedial (im) part which seems to correspond with the MFB of the rodents and passes from the lateral wall of the third ventricle to go to the lateral part of the hypothalamus (Coenen et al., 2012). A superolateral (sl) part leaves the VTA to ascend to the anterior limb of the internal capsule (ALIC) (Coenen et al., 2012). This part of the MFB connects to the accumbens nucleus (NAC) and the ventral striatum (Coenen et al., 2012). All species seem to have one thing in common: MFB connects the VTA with the lateral hypothalamus. The tract is composed of shorter and longer ascending and descending tributaries (Coenen et al., 2009a). They enter and leave this WMT as small fascicles (Coenen et al., 2009a). Physicians suggest that parts of the MFB might reach as far as the olfactory tubercle of the frontal lobe (Coenen et al., 2009a). Many of these stations like cingulated cortex (Brodmann area Cg25; Mayberg, 1997), ALIC (Greenberg et al., 2008), NAC (Berton and Nestler, 2006) which are connected by the MFB represent targets for DBS in patients with psychiatric disorders. DBS was first approved by the FDA in 1997 for patients with essential tremor (Karas et al., 2013). This technique was later approved for use in patients with PD, dystonia, or patients with major depression (MD) (Karas et al., 2013; Furlanetti et al., 2015). DBS delivers electrical pulses which are variable in amplitude, pulse width, and frequency, through permanently implanted electrodes (Perlmutter and Mink, 2006; Mädler and Coenen, 2012). The mechanism of DBS might consist in activation of a structure, inhibition of a structure, a combination of both, or disruption of a pathological oscillation (Karas et al., 2013). One of the major advantages of this treatment consists in the reversibility (Awan et al., 2009). The use of DBS presents an option in the treatment of MD which is reversible and at the same time adjustable (Morishita et al., 2014). MD is a common disease which has a prevalence in lifetime of 15–20% and might lead to disability of a person worldwide (Kupfer et al., 2012; Taghva et al., 2013). One of the main symptoms in patients with MD is their inability to experience pleasure (Rea et al., 2014). As the MFB seems to be a prominent structure of the reward system, different cerebral lesions like tumors (glioblastoma, meningioma, or metastasis) or bleeding might affect our behavior in a negative way by means of destroying, infiltrating, or pushing away the fibers of this tract. Glioblastoma is the most aggressive and lethal primary brain tumor (Norden et al., 2009). Its therapy consists in maximal tumor resection which is followed by radiation and chemotherapy (Cohen et al., 2009). About 25% of the patients with systemic cancer suffer from brain metastasis. The treatment consists either in surgical removal or radiation and chemotherapy, always depending on the primary cancer (Curry et al., 2005). Both lesions might affect the fibers of the MFB negatively and might be responsible for personality changes in our patients if they affect the MFB. Using special MRI modalities like DTI might help physicians distinguish between glioblatoma and metastasis (Chen et al., 2012) and furthermore be used during surgery when these lesions border the MFB in order to avoid a destruction of the tract by surgeons.

Our aim was to portray the MFB in patients with different pathologies in order to see how this WMT was affected by different lesions and whether there were clinical symptoms which could be attributed to lesions in this WMT. Furthermore, we wanted to portray the MFB in patients who would undergo a DBS in order to draw a comparison between the MFB in different patients with different diseases. An important issue was the portrayal of the MFB in relation to known and established deep brain stimulation targets like the STN. Neurosurgeons might be confronted with unwanted side effects during deep brain stimulation due to the proximity of our target and the MFB. Another important issue was to see whether there was a correlation between MFB lesions and the clinic of our patients.

## Materials and methods

### Patient data

DTI was performed according to the guidelines and the ethical standards of the Centre Hospitalier de Luxembourg. The research protocol was approved by the Institutional Review Boards of the Centre Hospitalier de Luxembourg and was conducted according to the principles expressed in the Declaration of Helsinki. All participants provided written informed consent. We performed DTI of the brain between 2012 and 2015 on 15 consecutive patients. There were nine men and six women (Table 1 summarizes the details). Patients can be divided into two groups. On the one hand we visualized the MFB on nine patients who would eventually undergo a DBS. Eight of these patients suffered from PD while one patient suffered from Multiple sclerosis. The other group consists of six patients. These were patients suffering from different cerebral lesions which were situated in the frontal lobe or in the vicinity of the frontal lobe. These were 2 metastasis, 2 meningiomas, 1 glioblastoma, and 1 cerebral bleeding which was mainly situated in the external capsula, but however, affected at a certain extent the frontal lobe as well. The mean age was 59 years. The youngest patient was 39 years old while the oldest patient was 79 years old. The individual age is specified in Table 2.

### Data acquisition

We used a 3DT1-sequence for the navigation. Furthermore T2- and DTI- sequences were performed. The FOV was 200 × 200 mm^2^, slice thickness 2 mm, and an acquisition matrix of 96 × 96 yielding nearly isotropic voxels of 2 × 2 × 2 mm. 3-Tesla-MRI was carried out strictly axial using 32 gradient directions and one b0-image. We used Echo-Planar-Imaging (EPI) and ASSET parallel imaging with an acceleration factor of 2. *b*-value was 800 s/mm^2^. The maximal angle was 50°. FA start value of 0.10 and ADC stop value of 0.20 mm^2^/s were the parameters used for tractography. Additional scanning time was less than 9 min.

### DTI-preparation

After having performed a 3DT1-, T2, and DTI-sequences we transferred the data to digital imaging and communications in medicine (DICOM). The next step consists in the transfer of the data to a surgical navigation program. This step has been previously widely described in one of our previous articles (Hana et al., 2014). If we are dealing with a patient who needs a DBS, we perform the fibertracking by using two ROIs. The size of the ROI was 1 cm One ROI is put in the frontal lobe, in the medial orbitofrontal cortex, while the localization of the other ROI is in the midbrain in the ventral tegmental area. In a patient with a cerebral lesion like glioblastoma or cerebral hemorrhage, there might be necessary to segment the lesion and an eventual edema which is surrounding this lesion too (Hana et al., 2014). When the site was invaded by an edema or by a lesion then we include parts of it in the ROI. Afterwards the images are imported to operating room from the navigation system and finally they can be integrated in the operation.

### DTI-limitations

It can only resolve single fiber directions but it can't depict crossing or kissing fibers (Zhang et al., 2013). Partial volume effect, like contamination with cerebrospinal fluid, is another important limitation (Zhang et al., 2013). Small tracts with different directions might not be identified. A similar effect is to be expected with an edema caused by a cerebral lesion like tumor (Wang et al., 2011). An edema infiltrates the brain tissue and hinders the portrayal of WMT in these areas. The actual size of fiber bundles might not be correctly presented even though certain parts of these tracts are identified (Kinoshita et al., 2005). Thus, WMT like the MFB might be underestimated in the presence of edema. Further limitations consist in the brain shift which remains an area that is still unsolved(Abdullah et al., 2013). Another important issue is the fact that DTI doesn't determine with accuracy the starting point or the end point of a WMT, producing multiple artifacts, and false tracts (Le Bihan et al., 2006). The possibility of false positive or false negative tracts should be kept in mind always.

## Results

**Figure 1:**
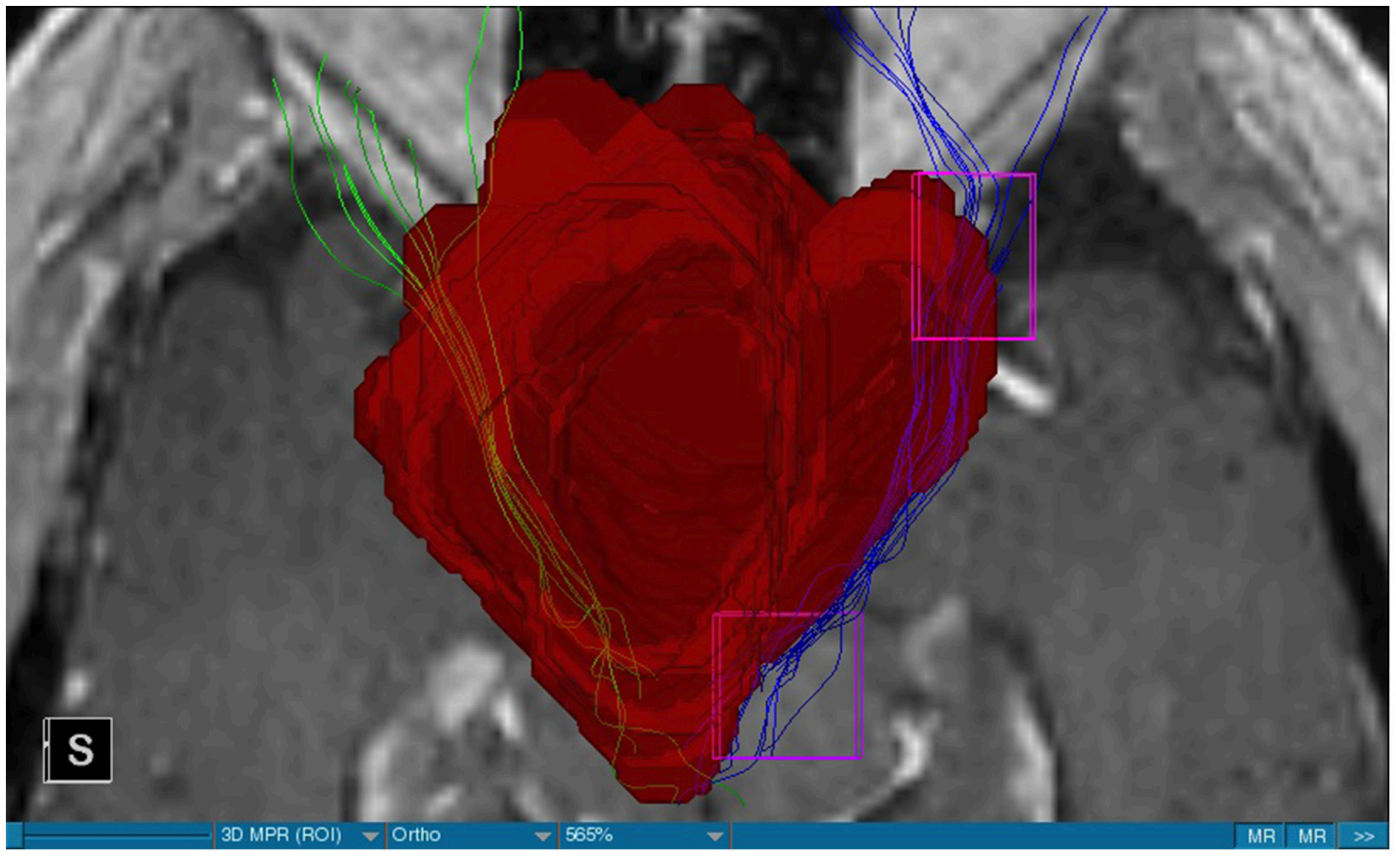
**MRI axial view, T1, red: Glioblastoma, blue: right MFB, green: left MFB**.

**Figure 2:**
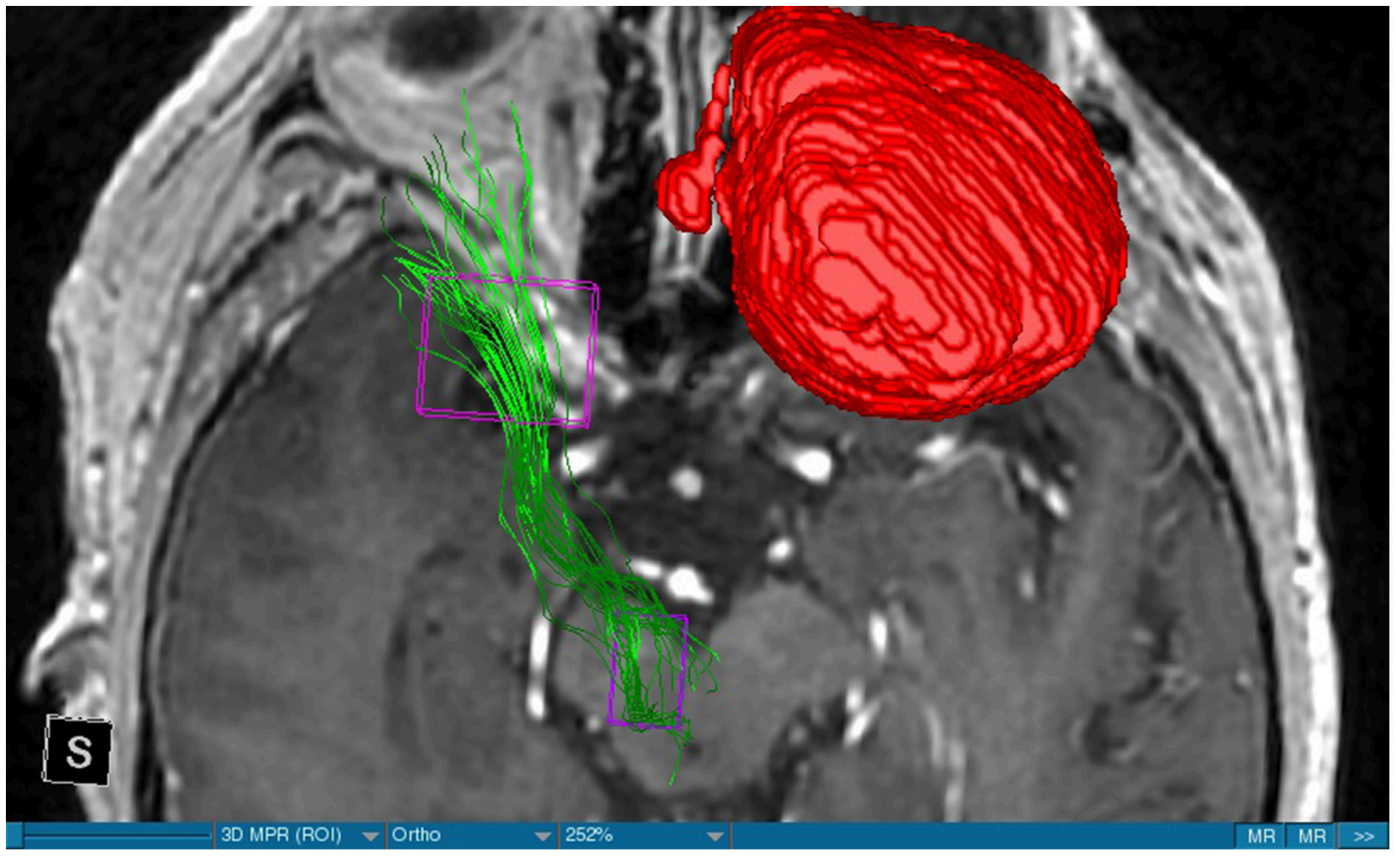
**MRI axial view, T1, red: Meningioma, green: left MFB, right MFB is missing**.

**Figure 3:**
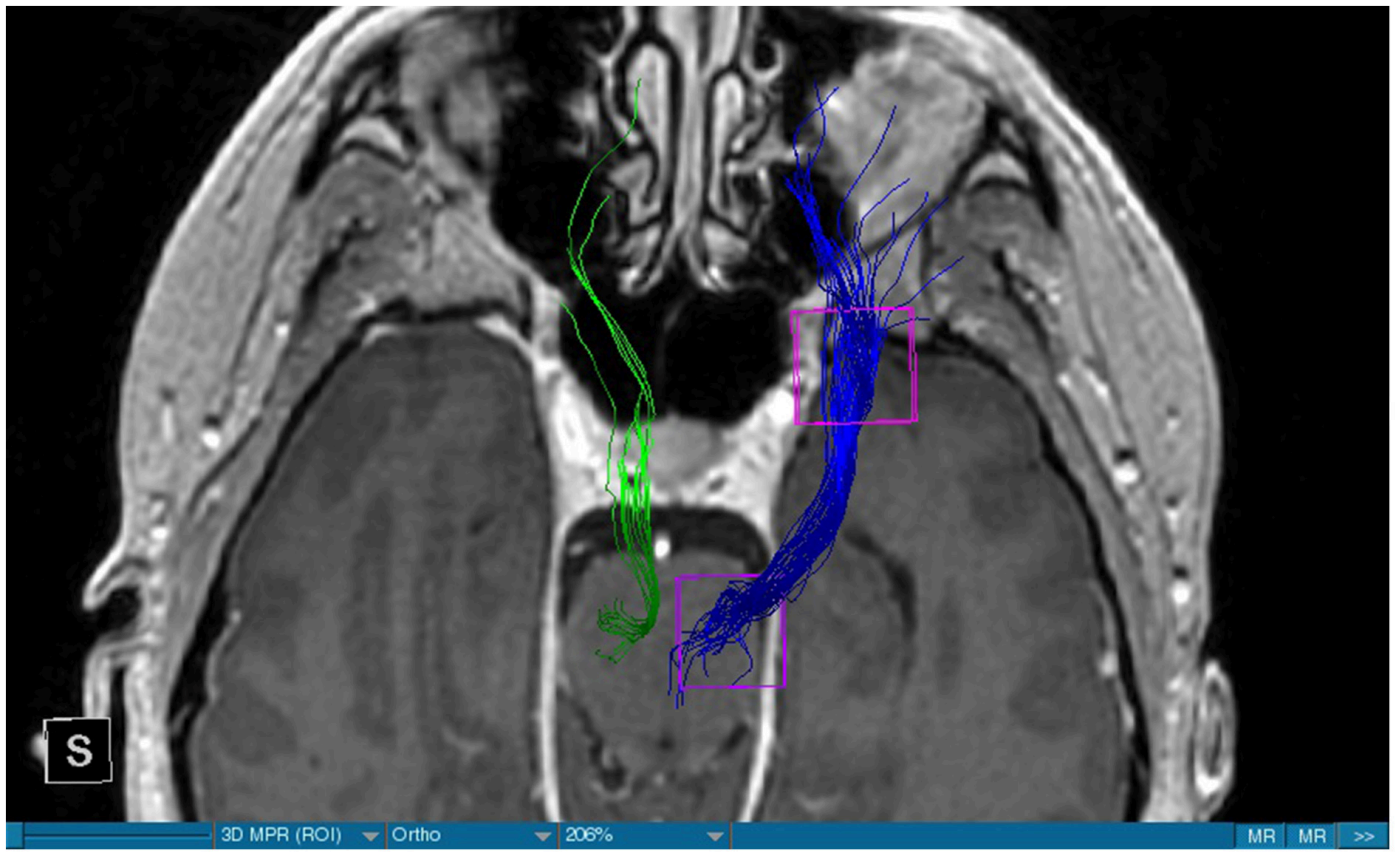
**MRI axial view, T1, DBS, blue: right MFB, green: left MFB**.

**Figure 4:**
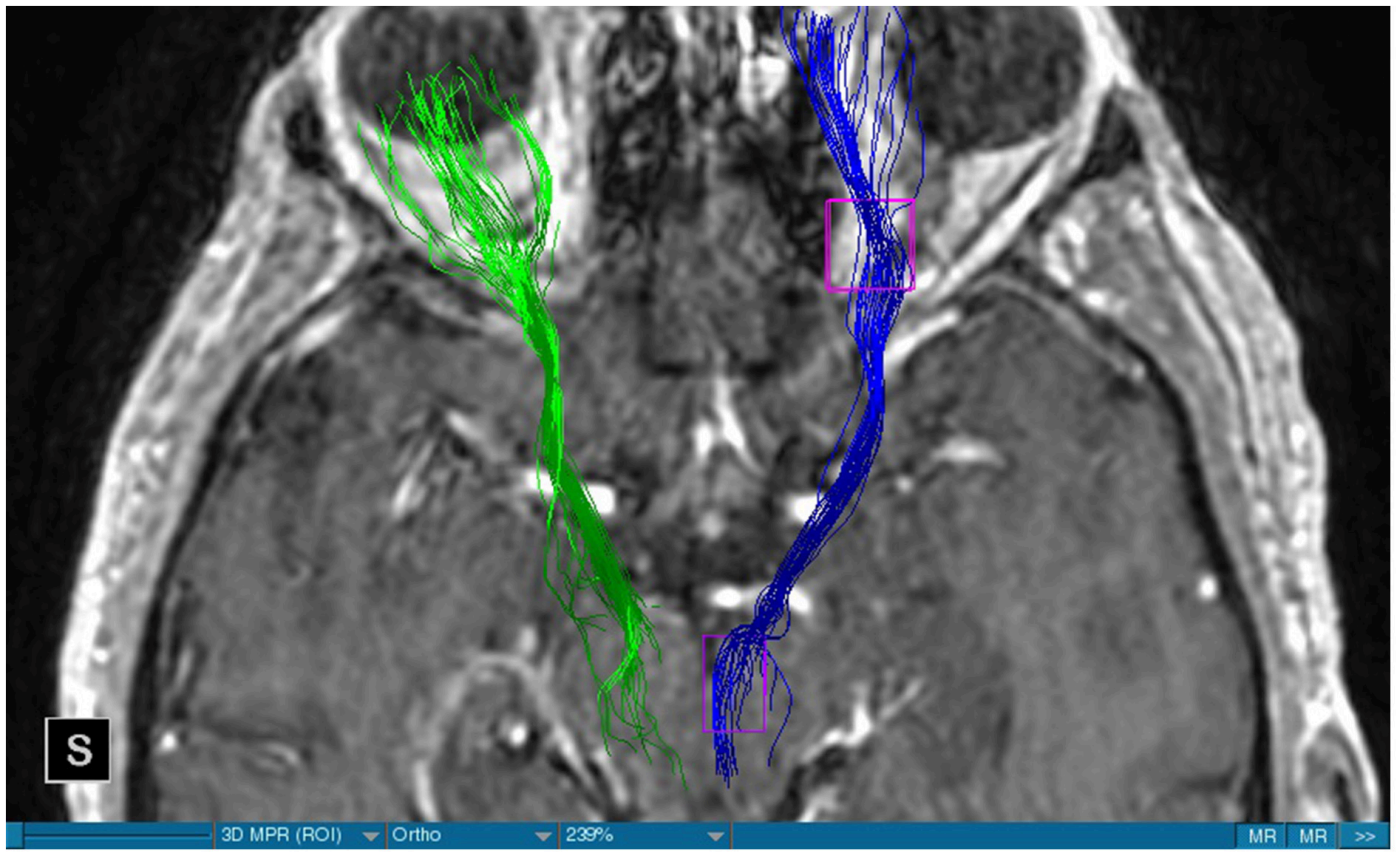
**MRI axial view, T1, DBS, blue: right MFB, green: left MFB**.

**Figure 5:**
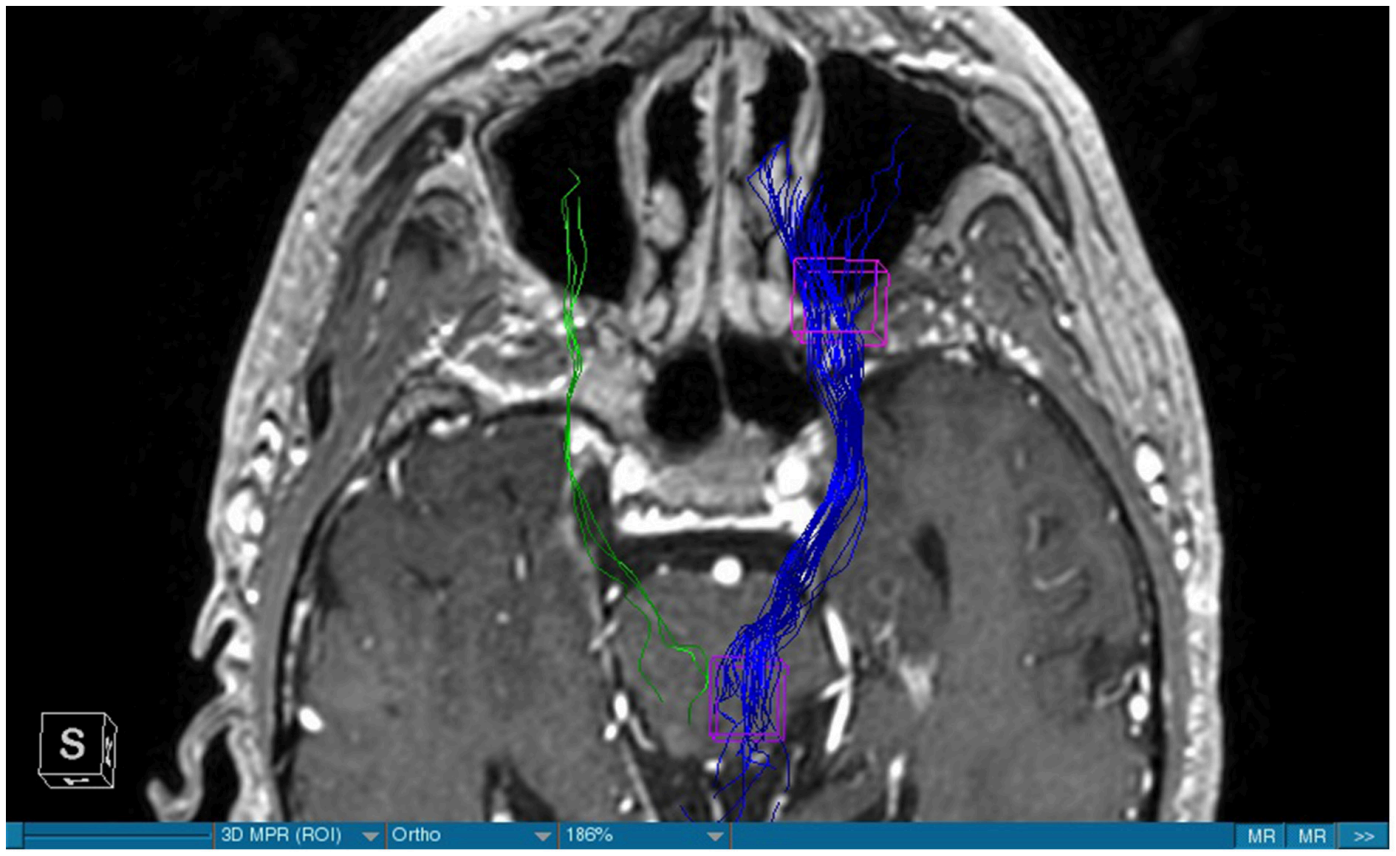
**MRI axial view, T1, DBS, blue: right MFB, green: left MFB**.

**Figure 6:**
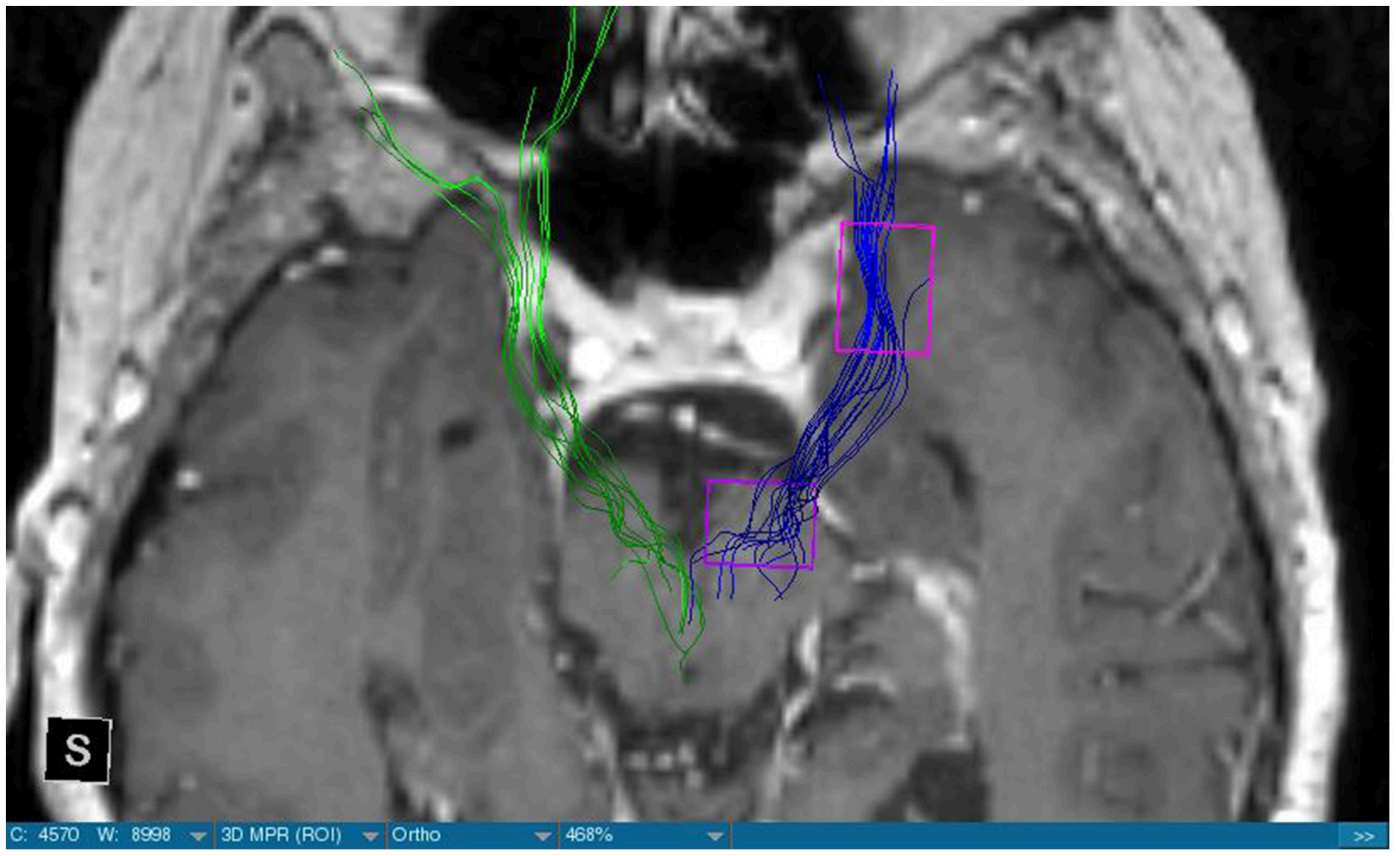
**MRI axial view, T1, DBS, blue: right MFB, green: left MFB**.

**Figure 7:**
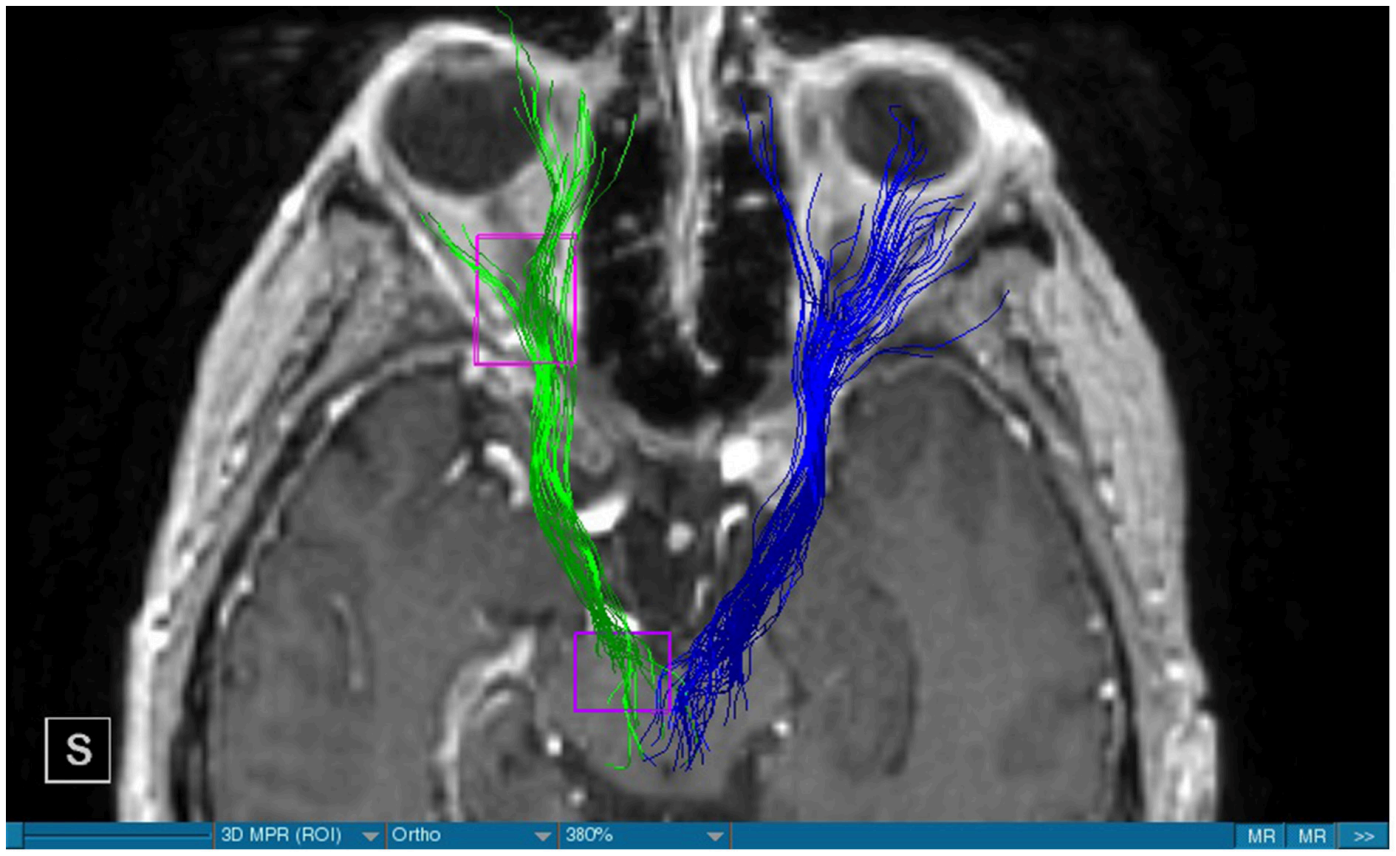
**MRI axial view, T1, DBS, blue: right MFB, green: left MFB**.

**Figure 8:**
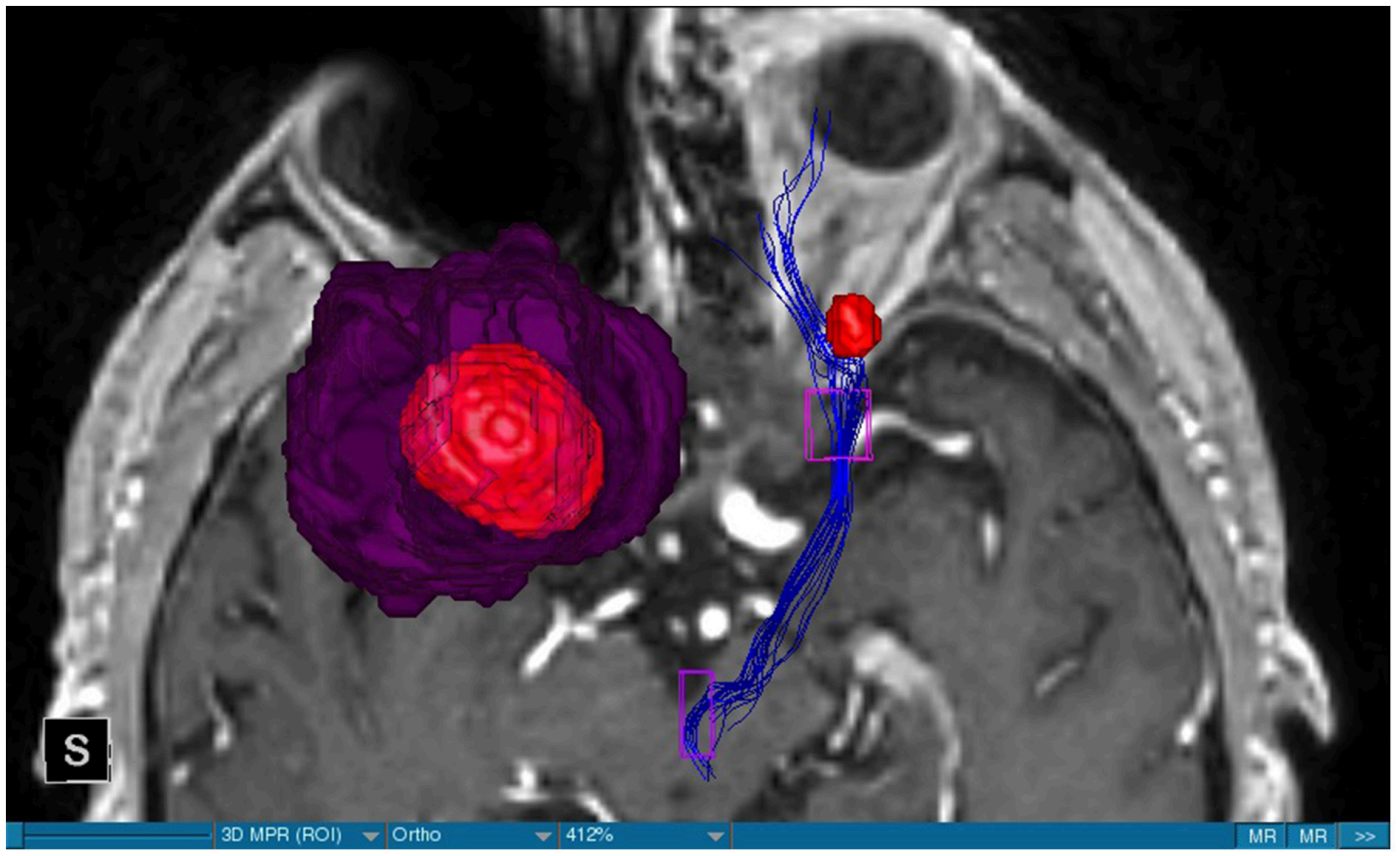
**MRI axial view, T1, red: Metastasis, purple: edema, blue: right MFB, left MFB is missing**.

We were able to portray the MFB in all our patients. In 12 of our patients the MFB could be visualized bilaterally and in the remaining three patients we depicted the MFB on one side. The MFB was missing on the ipsilateral side of the lesion. These were 2 meningiomas and 1 metastasis. In one case the lesion was surrounded by edema. We put our ROI as mentioned above in the VTA and in the medial orbitofrontal cortex. If one of these parts of the brain was influenced by a lesion e.g., edema we segmented the edema and included a part of it in our ROI. We assume that the fibers which could be portrayed were indeed correct and for sure but we never forgot that other fibers might be there too which couldn't be depicted due to the lesion. One case showed the MFB running laterally of the glioblastoma which was situated in between of the two MFB (Figure 1). Here we have the MFB depicted in blue and green. What is striking here, is the fact that a good part of the fibers are identified despite the big tumoral lesion, however they seem to have a small volume in comparison with Figure 3 or Figure 7. This might be due to the tumor which has invaded or destructed them or due to the limitation of DTI to depict WMT next to cerebral lesions completely. With other words we are sure about the correctness of the depicted fibers, however, there might be other fibers which are destroyed or disrupted by this tumor which couldn't be depicted with the DTI. This fact should always be kept in mind when visualizing WMT by means of DTI. This patient was put under antidepressive treatment in the follow up. In this respect it might be possible that the fibers of both MFB were compromised. When a certain number of fibers isn't working then symptoms might appear. The fibers pass through the superior cerebellar penduncle. They ascend through the pons and pass through two important areas. One of them is the area posterior of the red nucleus and the other one is the periaqueductal gray. The tract reachs furthermore the lateral hypothalamus. Furthermore, its fibers run through the lateral wall of the third ventricle and are situated next to the anterior horn of the lateral ventricle The fibers of this WMT are localized under the thalamus before they border the anterior limb of the internal capsule. The STN seems to be situated laterally and posteriorly of the MFB. Partially the MFB is directed latero-caudally at the level of the substantia nigra. The MFB ran from the VTA to the NAC, the medial and lateral orbitofrontal contex, and the dorsolateral prefrontal cortex. Ultimately it seems that the fibers reach out to the olfactory bulb. The MFB was situated cranially to the optic chiasm and the optical tract in his part concerning the frontal lobe. In Figure 2 we depicted the MFB on the healthy side only. On the ipsilateral side of the metastasis we couldn't portray the MFB. The patient however wasn't depressive and didn't need any treatment in this direction later on. This can be due to the presence of a good developed MFB on the contralateral side which is able to take over the task alone. Figures 3, 4 can show the fibers without being compromised by a lesion. Here we can see precisely the size of the ROI and their location. Figure 5 describes the case in which fibers of the MFB can differ in volume in one and the same patient without being compromised by a lesion. These differences can appear between the right and the left MFB in the same patient or there can be interindividual differences without any presence of special symptoms. The latter case can be seen if we compare Figure 6 with Figure 7. In Figure 8 we recognize the edema which is surrounding the tumor, in this case a metastasis. We can deduce that a lesion like edema can hinder the depiction of WMT by means of DTI. In Figure 2 there was a lesion without edema which hindered the portrayal of the MFB. In this case we have the presence of the two. We didn't remark any differences in our patients concerning the fractional anisotropy. We have to say that none of our patients sufferend from a MD. Based on the Hamilton scale for depression no one from our patients fulfilled the criteria for MD. We have to admit however that when you announce to a patient a diagnosis like brain cancer, he might feel hopeless or sad but this is only one of the criteria in this scale and it is something expected. None of our patients needed a psychiatric surveillance and they were stable during their whole stay in the hospital.

**Table 1:** **Demographics of all patients**.

	**All**	**DBS**	**Other lesions**	**Men**	**Women**	**MFB depiction bilaterally**	**MFB depiction unilaterally**	**Mean age**	**Lesion**
Patient	15	9	6	9	6	12	3	59 y	2 Metastasis
									2 Meningiomas
									1 Bleeding
									1 Glioblastoma

**Table 2:** **Exact age of the patients**.

**Pat. 1**	**Pat. 2**	**Pat. 3**	**Pat. 4**	**Pat. 5**	**Pat. 6**	**Pat. 7**	**Pat. 8**	**Pat. 9**	**Pat. 10**	**Pat. 11**	**Pat. 12**	**Pat. 13**	**Pat. 14**	**Pat. 15**
53 y	79 y	44 y	65 y	64 y	64 y	69 y	61 y	69 y	71 y	44 y	43 y	54 y	39 y	67 y

## Discussion

The MFB is a WMT of the forebrain which has been keeping physicians occupied since the second half of the Nineteenth century. It was first described by Ganser in 1882 and later on other physicians tried to enlighten this WMT (Nieuwenhuys et al., 1982). The importance of the MFB becomes more evident when we know about its role as a key structure of the reward system (Bracht et al., 2014). The reward circuit is very important for developing motivated behaviors (Haber and Knutson, 2010). Significant components of this network are the anterior cingulated cortex, the orbital prefrontal cortex, the ventral striatum, the ventral pallidum, the dopamine neurons in the midbrain, NAC, and the VTA (Haber and Knutson, 2010). Neuroimaging studies like functional magnetic resonance imaging, positron emission tomography or DTI have been used to elucidate these areas more precisely (Wacker et al., 2009). DTI is used by physicians to depict WMT in healthy and non-healthy patients *in vivo* among others the MFB (Mesulam, 2005; Stadlbauer et al., 2006; Ciccarelli et al., 2008). This WMT has been identified in rodents and humans with partially big differences. Whereas in humans this WMT is described as a bipartite structure, in rodents this tract is located mainly in the lateral wall of the hypothalamus as a steak-like structure (Coenen et al., 2009a, 2012). However, despite these differences this tract connects the VTA with the lateral hypothalamus in all species. Our results confirm furthermore that this WMT borders various important structures of the brain like the hypothalamus, or the STN. These results correspond with the findings of other groups (Coenen et al., 2009a, 2012). Alteration of WMT who are responsible for the reward system might be responsible for MD (Coenen et al., 2009b). These alterations might be caused by inflammatory diseases or by different cerebral lesions. Tumors or edema might compromise the FA-values. In the same time they might be responsible for changes in the behavior of the patients. In our patient with a giant meningioma the first symptoms had predominantly a behavioral character. Her temper changed from absolutely calm to autoaggression. In her case we couldn't portray the MFB on the ipsilateral side of the tumor. In all our patients we didn't remark any special FA-reductions (Figures 1, 2, 8). Especially FA-reductions in the slMFB have been associated with anhedonia in patients with acute depression (Bracht et al., 2015b). Lower FA-values in the slMFB seem to be associated with melancholic depression also (Bracht et al., 2015a). However, there is a negative correlation between the hedonic capacity and the mean FA of the slMFB for patients in remission or for those who were never depressive (Bracht et al., 2015a). Lower FA in the left slMFB seems to be associated with more pronounced capacity to derive pleasure in patients who were never depressive or in patients in remission (Bracht et al., 2015a). Alterations have been found also in other structures which incorporate the slMFB (Bracht et al., 2015a): Reduction in FA-values has been found in melancholic patients too in VTA-orbitofrontal cortex connections too (Bracht et al., 2014). Besides that these authors found out that the higher the depression the lower the FA in melancholic patients (Bracht et al., 2014). These alterations seem to concern the slMFB. Alterations in this area of the brain might contribute to the severity of the disease (Bracht et al., 2014). Keeping in mind that tumors don't respect borders or WMT, we have to say that in their presence we might find the same alterations. Other neurodegenerative diseases might reveal the same results. In those cases we have to try to find out how big is the influence of the MD and how big that of the other illness. However, the authors found no correlation between hedonic tone and imMFB (Bracht et al., 2015a). This might lead to the assumption that slMFB is more important in cases of hedonia or anhedonia than the imMFB. The importance of the slMFB for patients with depression has been highlighted in another study. According to them, this part of the MFB is a very important station of reward and mood (Schoene-Bake et al., 2010). Another group came also to the conclusion that stimulating the slMFB might be advantageous in patients with depression (Schlaepfer et al., 2013). In a study using deterministic methods to identify the slMFB they saw positive results in the treatment of patients with MD by means of bilateral DBS in slMFB (Schlaepfer et al., 2013). We have to say that our patients weren't depressive and only the patient in Figure 1 needed antidepressive medication in his follow-up. So according to these findings a reduction of the FA wasn't expected and ultimately wasn't present. The authors describe furthermore no changes in the mean FA of the slMFB in patients with remission and those who never were depressive (Bracht et al., 2015a,b). These changes are no markers however they seem to be state-dependent as they disappear when the patients are in remission (Bracht et al., 2015a). If there is no cerebral lesion we might tend to attribute these changes in FA to the MD but in the presence of a tumor or edema FA-value might be compromised too. In that case it might be difficult for physicians to attribute certain changes to MD, to the tumor, or to the edema. A comparison between patients without MD but with a tumor and those with MD but without a tumor would be necessary to see how the FA-value is influenced due to a certain situation in order to draw a conclusion. Furthermore, the MFB is confirmed as the central pathway of the reward system (Bracht et al., 2015a). Coenen et al. have portrayed two different branches of the MFB in humans. One of them is the medial branch which connects the lateral hypothalamus with the reticular formation of the brain stem and the lateral branch which projects to several centers of the brain like the subcoeruleus nucleus or the substantia nigra pars compacta (Coenen et al., 2009a). Both branches form a “W” shape in the coronal image according a study which was published recently, whereby the imMFB presents the middle portion of the “W,” and the lateral portions of the “W” consist of the slMFB (Cho et al., 2015). Although the findings of this study were mostly comparable with the results of other physicians there was however a new insight in the relation between the anterior thalamic radiation (ATR) and the slMFB. The authors describe the position of the ATR superior to the slMFB lateral of this branch when entering the ALIC although it is situated medially before its entry (Cho et al., 2015). This knowledge might be of substantial importance for neurosurgeons who perform DBS in patients with MD. Stimulation of the slMFB might be beneficiary in patients with depression (Coenen et al., 2009a), whereas stimulation of the ATR is thought to have an opposing effect (Cho et al., 2015), therefore it is important for physicians to know the exact location of these tracts in order to avoid stimulation of the wrong WMT as well as to avoid a co-stimulation of both tracts. These results were obtained using a 7-Tesla-MRI and most of them were consistent with the results obtained by other authors before who were using a 3-Tesla-MRI (Coenen et al., 2012). One of the reasons for the discrepancy might be the fact that DTI has difficulties portraying kissing or crossing fibers in a satisfactory way (Wakana et al., 2004). For the depiction of the MFB there exist many possibilities which have lead to more or less the same results. Therefore, one group used for the portrayal of the MFB two ROI which were placed in the following way: one in the ventral midbrain and the second one included the NAC and the lateral hypothalamus (Coenen et al., 2009a). The lateral hypothalamus is an important part in the rodents MFB and corresponds more or less with the human imMFB. In another study the group around Coenen identified the MFB by using only one ROI which was placed in the VTA (Coenen et al., 2012). There was no difference found in the course of the MFB in comparison of the two different tracking methods. Another more recent study describes the identification of the MFB by using three ROI in patients who underwent DBS. This group placed the ROI in the superior cerebellar peduncle, VTA, and the dorsal raphe nucleus which present stations in the course of the MFB (Anthofer et al., 2015). slMFB was consistently depicted by their method whereas the imMFB could only be inconsistently portrayed (Anthofer et al., 2015). They depicted the MFB in 20 out of 22 hemispheres. In the remaining two they couldn't depict it. In our group we weren't able to portray the MFB in three hemispheres. These hemispheres were invaded by a lesion. In all our DBS patient we were able to portray the MFB on both sides. However, there aren't yet standardized parameters for the portrayal of the MFB. Thus, studies described here are performed on a small group of patients. This fact should be taken into account. DTI sequences with a higher resolution might be more helpful in this case.

Limitations concerning the visualization of WMT by means of DTI consist in the depiction of branched or merged fibers (Zhang et al., 2013). It seems to be very difficult to portray WMT like the MFB in regions which are invaded by cerebral lesion, particularly when these lesions are surrounded by a cerebral peritumoral edema (Zhang et al., 2013). In our group of patients we didn't experience any troubles in portraying the MFB in patients who were planned for a DBS (patients without cerebral lesions), however, we weren't able to portray this WMT in patients with cerebral lesions, which were 2 meningiomas and 1 metastasis. On one hand one can argue the MFB was damaged by the lesion, in these cases by the meningiomas or the metastasis, on the other hand the absence of the MFB on the side of the lesion might be attributed to the difficulties of DTI in depicting this tract in the presence of cerebral lesions. In our glioblastoma patient of Figure 1 we were able to portray the MFB bilaterally but we have to keep in mind that there might be other fibers which weren't identified due to the lesion. If we compare Figure 1 with Figure 7 we remark a great interindividual difference between the two. The size and the volume of the fibers in Figure 7 is much bigger than in Figure 1. In Figures 2, 8 the tract wasn't portrayed on the ipsilateral side of the brain tumor. When the depiction of a tract fails, it may not always mean that there is no tract at all (Hana et al., 2015). Cerebral lesions might affect WMT in different ways by invading, displacing, or disrupting them (Abdullah et al., 2013). In the case of the MFB they might compromise the mood of patients. In our DBS-patients we didn‘experience any kind of a tendency to depression, however, in our glioblastoma patient there was the necessity of prescribing antidepressive medication as part of the further follow-up. In his case the tumor was localized between the two MFB and it concerned both frontal lobes (Figure 1). We should keep in mind that such medication might be necessary in many tumor patients and one might say one case is not very specific however when the MFB is concerned this might be a starting point. As the DTI doesn't say anything about the function of WMT we can't say that the fibers we are watching do perform their task correctly. Thus, a patient may have symptoms despite the MFB being identified. We might be allowed to say however that when the tract is damaged and the patients show symptoms which may be attributed to the functions of the tract that our imagery findings correspond with the clinics. Another case is that of a patient which was operated from a giant frontal meningioma. The MFB wasn't visible on the side of the tumor. This might be on one hand due to a limitation of the DTI or because the tumor destroyed the WMT. However, one of the main symptoms was the lack of enthusiasm. Again this might occur in many tumors but here it coincides with a non-visualization of the MFB. The presence of a cerebral lesion doesn't compulsorily mean that the depiction of the WMT won't be successful. We were able to depict the MFB on both sides e.g., in a patient with glioblastoma (Figure 1). Furthermore, DTI can't inform the physicians about the starting or the ending point of the WMT (Hana et al., 2015) which might lead to artifacts. Another important limitation of DTI which consist in the brain shift during surgery is an important issue we need to deal with (Abdullah et al., 2013). After opening the dura mater and after manipulation in the brain e.g., by removing a lesion or by putting an electrode inside the brain we don't have the same conditions we used to have before surgery. One can argue that for the resection of tumors in non-eloquent areas the results might be satisfactory but we have to keep in mind that one or two millimeters next to the MFB might be destructive for this WMT during tumor surgery. During DBS this distance might mean another WMT or another target point. By a localization of the MFB medially to the STN that would mean a stimulation of the STN instead of the MFB and vice-versa. In this case we might expect side-effects in our patients. This risk of costimulating the slMFB during DBS of the STN was described by Coenen et al. in another work (Coenen et al., 2009a). They presumed that acute hypomania during STN DBS might occur due to a unilateral and left sided activation of the imMFB (Coenen et al., 2009a). This part of the MFB is more associated with wanting while the slMFB is associated with liking (Coenen et al., 2009a). As our results furthermore show, the slMFB is situated in the ALIC. If we stimulate this part of the brain we might have an eventual unwanted motoric involvement in our patients. Other structures which might be affected during surgery in patients with DBS include the red nucleus or parts of the hypothalamus. In our results both parts of the MFB were localized superiorly to the visual pathway. Coenen et al. have reported that quality of life in patients with PD might be negatively influenced by depression during DBS of the STN. This seemed to happen when the electrode was located anteriorly, inferiorly and medially (Coenen et al., 2009b). As the MFB seems to be situated anteriorly and medially of the STN this seems to be obvious, however, it seems not to be clear why we have this influence inferiorly. As it is a part of the reward circuitry we already mentioned above, this WMT might be eventually used for DBS in patients suffering from MD. Keeping in mind all the important cerebral structures we mentioned above which we should avoid to stimulate, there seems to be no consensus as which part of the MFB is the best target point in patients suffering from MD. There have been many suggestions of target points which are partly connected by the MFB like the NAC, cingulate cortex, or the ventral internal capsule (Döbrössy et al., 2015). In animal models there have been studies observing DBS in STN or the NAC. The bilateral stimulation of these targets reduced addiction (Döbrössy et al., 2015). A recent study has shown that a stimulation of the MFB but not a stimulation of the Cg25 interacts with the reward system in rats (Edemann-Callesen et al., 2015). Another important issue is to know how many fibers need to be stimulated in order to achieve satisfactory results. Even if we stimulate the right structure, we might have disappointing results because of the lack of a sufficient volume of fibers wasn't stimulated. Concerning depression, different physicians have used different target points with different scores which makes it more difficult to draw a conclusion. Some of the target points include the NAC, the subcallosal cingulate gyrus, and experimentally the VTA (Döbrössy et al., 2015). All these structures have a common ground. Many of their afferent or efferent connections pass through the MFB (Döbrössy et al., 2015). The stimulation of these targets seems to reduce depression at a certain extent (Döbrössy et al., 2015). These results contradict old stimulation studies observed in rats that showed MFB stimulation was detrimental to the animals' health (Olds, 1963). Up to date it is very little known about the reaction of individuals when stimulating the MFB directly. Although there are differences between the studies conducted then and now, this shows however that a certain risk is always present. Before patients are approved for DBS e.g., in PD there has to be a reliability that they aren't going to commit suicide. All these facts need to be taken into account before operating on such a patient. This risk concerns however all patients who undergo the procedure of a DBS and not only those who suffer from depression. Schlaepfer et al. confirm the positive effect of DBS in patients with depression in targets like NAC but on a recent study with seven patients which was conducted by them, they obtained faster results, within days, by stimulating the slMFB bilaterally (Schlaepfer et al., 2013). Bilateral stimulation of the slMFB seems to be mandatory for positive results. On the other hand the ATR seems to mediate distress and sadness in humans (Coenen et al., 2012). This WMT runs in the proximity of the MFB and seems to be its counterpart. A balance between these two WMT seems to be mandatory for the well-being of a person. During DBS surgery, physicians should try to avoid this tract. And therefore the correct knowledge of its localization is mandatory in order to avoid unnecessary side effects. Satisfactory results are to be achieved when we portray the MFB and ATR by means of DTI in the same time. The use of DTI allows the depiction of the MFB and other WMTs in patients with cerebral lesions as well as in patients who will undergo a DBS. Its portrayal is easy and feasible for everyday routine. Nevertheless, a neurosurgeon needs to keep in mind the anatomy of the MFB and that of other structures in the surrounding for a successful surgery. The limitations of DTI however shouldn't be neglected. Based on our findings of this small group we have to say that depiction of the MFB is often compromised when other lesions are in the proximity of this tract but the identification of the MFB is still possible for every day routine. Tracts might be missing like in three of our patients or they can be influenced in other forms like invasion, infiltration, or displacement. However, patients don't seem to have symptoms when only one of the MFB is absent (Figures 2, 8). In this case the MFB of the other side might take over the task. The need for antidepressive medication might become necessary in the follow-up of patients with unilateral portrayal of the MFB (Figure 1). However, this can be an effect of the disease also. MFB can differ in its volume in one patient between the two hemispheres or it can be different between two different people without being influenced by some other lesion. Patients with cerebral lesions tend to have a less developed MFB than those without lesions. This doesn't mean that symptoms in such cases are present automatically. DTI is a tool that gives further insight in this tract but we should keep in mind that there are limitations which must be taken into account.

### Conflict of interest statement

The authors declare that the research was conducted in the absence of any commercial or financial relationships that could be construed as a potential conflict of interest.

## References

[B1] Abdullah K. G. Lubelski D. Nucifora P. G. Brem S. (2013). Use of diffusion tensor imaging in glioma resection. Neurosurg. Focus. 34:E1. 10.3171/2013.1.FOCUS12412 23544405

[B2] Anthofer J. M. Steib K. Fellner C. Lange M. Brawanski A. Schlaier J. (2015). DTI-based deterministic fibre tracking of the medial forebrain bundle. Acta Neurochir (Wien). 157, 469–477. 10.1007/s00701-014-2335-y 25585836

[B3] Awan N. R. Lozano A. Hamani C. (2009). Deep brain stimulation: current and future perspectives. Neurosurg. Focus. 27:E2. 10.3171/2009.4.FOCUS0982 19569890

[B4] Basser P. J. Mattiello J. LeBihan D. (1994). MR diffusion tensor spectroscopy and imaging. Biophys. J. 66, 259–267. 10.1016/S0006-3495(94)80775-1 8130344 PMC1275686

[B5] Berton O. Nestler E. J. (2006). New approaches to antidepressant drug discovery: beyond monoamines. Nat. Rev. Neurosci. 7, 137–151. 10.1038/nrn1846 16429123

[B6] Bracht T. Doidge A. N. Keedwell P. A. Jones D. K. (2015a). Hedonic tone is associated with left supero-lateral medial forebrain bundle microstructure. Psychol. Med. 45, 865–874. 10.1017/S0033291714001949 25124530 PMC4413785

[B7] Bracht T. Horn H. Strik W. Federspiel A. Schnell S. Höfle O.. (2014). White matter microstructure alterations of the medial forebrain bundle in melancholic depression. J. Affect. Disord. 155, 186–193. 10.1016/j.jad.2013.10.048 24252169

[B8] Bracht T. Jones D. K. Müller T. J. Wiest R. Walther S. (2015b). Limbic white matter microstructure plasticity reflects recovery from depression. J. Affect. Disord. 170, 143–149. 10.1016/j.jad.2014.08.031 25240841

[B9] Chen X. Z. Yin X. M. Ai L. Chen Q. Li S. W. Dai J. P. (2012). Differentiation between brain glioblastoma multiforme and solitary metastasis: qualitative and quantitative analysis based on routine MR imaging. Am. J. Neuroradiol. 33, 1907–1912. 10.3174/ajnr.A3106 22743640 PMC7964626

[B10] Cho Z. H. Law M. Chi J. G. Choi S. H. Park S. Y. Kammen A.. (2015). An anatomic review of thalamolimbic fiber tractography: ultra-high resolution direct visualization of thalamolimbic fibers anterior thalamic radiation, superolateral and inferomedial medial forebrain bundles, and newly identified septum pellucidum tract. World Neurosurg. 83, 54–61. 10.1016/j.wneu.2013.08.022 23973452

[B11] Ciccarelli O. Catani M. Johansen-Berg H. Clark C. Thompson A. (2008). Diffusion-based tractography in neurological disorders: concepts, applications, and future developments. Lancet Neurol. 7, 715–727. 10.1016/S1474-4422(08)70163-7 18635020

[B12] Coenen V. A. Honey C. R. Hurwitz T. Rahman A. A. McMaster J. Bürgel U. (2009a). Medial forebrain bundle stimulation as a pathophysiological mechanism for hypomania in subthalamic nucleus deep brain stimulation for Parkinson's disease. Neurosurgery 64, 1106–1114. 10.1227/01.NEU.0000345631.54446.06 19487890

[B13] Coenen V. A. Hurwitz T. Panksepp J. Mädler B. Honey C. R. (2009b). Medial forebrain bundle stimulation elicits psychotropic side effects in subthalamic nucleus deep brain stimulation for PD – new insights through diffusion tensor imaging. Akt Neurol. 36, P749 10.1055/s-0029-1238842

[B14] Coenen V. A. Panksepp J. Hurwitz T. A. Urbach H. Mädler B. (2012). Human medial forebrain bundle (MFB) and anterior thalamic radiation (ATR): imaging of two major subcortical pathways and the dynamic balance of opposite affects in understanding depression. J. Neuropsychiatry Clin. Neurosci. 24, 223–236. 10.1176/appi.neuropsych.11080180 22772671

[B15] Coenen V. A. Schlaepfer T. E. Maedler B. Panksepp J. (2011). Cross-species affective functions of the medial forebrain bundle-implications for the treatment of affective pain and depression in humans. Neurosci. Biobehav. Rev. 35, 1971–1981. 10.1016/j.neubiorev.2010.12.009 21184778

[B16] Cohen M. H. Shen Y. L. Keegan P. Pazdur R. (2009). FDA drug approval summary: bevacizumab (Avastin) as treatment of recurrent glioblastoma multiforme Oncologist 14, 1131–1138. 10.1634/theoncologist.2009-0121 19897538

[B17] Curry W. T. Jr. Cosgrove G. R. Hochberg F. H. Loeffler J. Zervas N. T. (2005). Stereotactic interstitial radiosurgery for cerebral metastases. J. Neurosurg. 103, 630–635. 10.3171/jns.2005.103.4.0630 16266044

[B18] Döbrössy M. D. Furlanetti L. L. Coenen V. A. (2015). Electrical stimulation of the medial forebrain bundle in pre-clinical studies of psychiatric disorders. Neurosci. Biobehav. Rev. 49, 32–42. 10.1016/j.neubiorev.2014.11.018 25498857

[B19] Edemann-Callesen H. Voget M. Empl L. Vogel M. Wieske F. Rummel J.. (2015). Medial forebrain bundle deep brain stimulation has symptom-specific anti-depressant effects in rats and as opposed to ventromedial prefrontal cortex stimulation interacts with the reward system. Brain Stimul. 8, 714–723. 10.1016/j.brs.2015.02.009 25819024

[B20] Furlanetti L. L. Döbrössy M. D. Aranda I. A. Coenen V. A. (2015). Feasibility and safety of continuous and chronic bilateral deep brain stimulation of the medial forebrain bundle in the naïve sprague-dawley rat. Behav. Neurol. 2015:256196. 10.1155/2015/256196 25960609 PMC4414266

[B21] Gálvez J. F. Keser Z. Mwangi B. Ghouse A. A. Fenoy A. J. Schulz P. E.. (2015). The medial forebrain bundle as a deep brain stimulation target for treatment resistant depression: a review of published data. Prog. Neuropsychopharmacol. Biol. Psychiatry. 58C, 59–70. 10.1016/j.pnpbp.2014.12.003 25530019

[B22] Greenberg B. D. Askland K. D. Carpenter L. L. (2008). The evolution of deep brain stimulation for neuropsychiatric disorders. Front. Biosci. 13:4638–4648. 10.2741/3029 18508535

[B23] Haber S. N. Knutson B. (2010). The reward circuit: linking primate anatomy and human imaging. Neuropsychopharmacology 35, 4–26. 10.1038/npp.2009.129 19812543 PMC3055449

[B24] Hana A. Dooms G. Boecher-Schwarz H. Hertel F. (2015). Diffusion tensor imaging - Arcuate fasciculus and the importance for the neurosurgeon. Clin. Neurol. Neurosurg. 132, 61–67. 10.1016/j.clineuro.2015.03.001 25795162

[B25] Hana A. Husch A. Gunness V. R. Berthold C. Hana A. Dooms G.. (2014). DTI of the visual pathway - white matter tracts and cerebral lesions. J. Vis. Exp. 10.3791/51946 25226557 PMC4828020

[B26] Karas P. J. Mikell C. B. Christian E. Liker M. A. Sheth S. A. (2013). Deep brain stimulation: a mechanistic and clinical update. Neurosurg. Focus 35, E1. 10.3171/2013.9.FOCUS13383 24175861

[B27] Kinoshita M. Yamada K. Hashimoto N. Kato A. Izumoto S. Baba T.. (2005). Fiber-tracking does not accurately estimate size of fiber bundle in pathological condition: initial neurosurgical experience using neuronavigation and subcortical white matter stimulation. Neuroimage 25, 424–429. 10.1016/j.neuroimage.2004.07.076 15784421

[B28] Kupfer D. J. Frank E. Phillips M. L. (2012). Major depressive disorder: new clinical, neurobiological, and treatment perspectives. Lancet 379, 1045–1055. 10.1016/S0140-6736(11)60602-8 22189047 PMC3397431

[B29] Le Bihan D. Poupon C. Amadon A. Lethimonnier F. (2006). Artifacts and pitfalls in diffusion MRI. J. Magn. Reson. Imaging 3, 478–488. 10.1002/jmri.20683 16897692

[B30] Mädler B. Coenen V. A. (2012). Explaining clinical effects of deep brain stimulation through simplified target-specific modeling of the volume of activated tissue. Am. J. Neuroradiol. 33, 1072–1080. 10.3174/ajnr.A2906 22300931 PMC8013266

[B31] Mayberg H. S. (1997). Limbic-cortical dysregulation: a proposed model of depression. J. Neuropsychiatry Clin. Neurosci. 9, 471–481. 10.1176/jnp.9.3.471 9276848

[B32] Mesulam M. (2005). Imaging connectivity in the human cerebral cortex: the next frontier? Ann. Neurol. 57, 5–7. 10.1002/ana.20368 15622538

[B33] Morishita T. Fayad S. M. Higuchi M. A. Nestor K. A. Foote K. D. (2014). Deep brain stimulation for treatment-resistant depression: systematic review of clinical outcomes. Neurotherapeutics 11, 475–484. 10.1007/s13311-014-0282-1 24867326 PMC4121451

[B34] Nieuwenhuys R. Geeraedts L. M. Veening J. G. (1982). The medial forebrain bundle of the rat. I. General introduction. J. Comp. Neurol. 206, 49–81. 10.1002/cne.902060106 6124562

[B35] Norden A. D. Drappatz J. Wen P. Y. (2009). Antiangiogenic therapies for high-grade glioma. Nat. Rev. Neurol. 5, 610–620. 10.1038/nrneurol.2009.159 19826401

[B36] Olds J. (1963). Self-stimulation experiments. Science 140, 218–220. 10.1126/science.140.3563.218 13939937

[B37] Perlmutter J. S. Mink J. W. (2006). Deep brain stimulation. Annu. Rev. Neurosci. 29, 229–257. 10.1146/annurev.neuro.29.051605.112824 16776585 PMC4518728

[B38] Rea E. Rummel J. Schmidt T. T. Hadar R. Heinz A. Mathé A. A.. (2014). Anti-anhedonic effect of deep brain stimulation of the prefrontal cortex and the dopaminergic reward system in a genetic rat model of depression: an intracranial self-stimulation paradigm study. Brain Stimul. 7, 21–28. 10.1016/j.brs.2013.09.002 24139146

[B39] Schlaepfer T. E. Bewernick B. H. Kayser S. Mädler B. Coenen V. A. (2013). Rapid effects of deep brain stimulation for treatment-resistant major depression. Biol. Psychiatry 73, 1204–1212. 10.1016/j.biopsych.2013.01.034 23562618

[B40] Schoene-Bake J. C. Parpaley Y. Weber B. Panksepp J. Hurwitz T. A. Coenen V. A. (2010). Tractographic analysis of historical lesion surgery for depression. Neuropsychopharmacology 35, 2553–2563. 10.1038/npp.2010.132 20736994 PMC3055575

[B41] Stadlbauer A. Nimsky C. Buslei R. Salomonowitz E. Hammen T. Buchfelder M.. (2006). Diffusion tensor imaging and optimized fiber tracking in glioma patients: histopathologic evaluation of tumor-invaded white matter structures. Neuroimage 34, 949–956. 10.1016/j.neuroimage.2006.08.051 17166744

[B42] Taghva A. S. Malone D. A. Rezai A. R. (2013). Deep brain stimulation for treatment-resistant depression. World Neurosurg. 80, S27.e17–24. 10.1016/j.wneu.2012.10.068 23111230

[B43] Wacker J. Dillon D. G. Pizzagalli D. A. (2009). The role of the nucleus accumbens and rostral anterior cingulate cortex in anhedonia: integration of resting EEG, fMRI, and volumetric techniques. Neuroimage 46, 327–337. 10.1016/j.neuroimage.2009.01.058 19457367 PMC2686061

[B44] Wakana S. Jiang H. Nagae-Poetscher L. M. van Zijl P. C. Mori S. (2004). Fiber tract-based atlas of human white matter anatomy. Radiology 230, 77–87. 10.1148/radiol.2301021640 14645885

[B45] Wang Y. Wang Q. Haldar J. P. Yeh F. C. Xie M. Sun P.. (2011). Quantification of increased cellularity during inflammatory demyelination. Brain 134(Pt 12), 3590–3601. 10.1093/brain/awr307 22171354 PMC3235568

[B46] Zhang H. Wang Y. Lu T. Qiu B. Tang Y. Ou S.. (2013). Differences between generalized q-sampling imaging and diffusion tensor imaging in the preoperative visualization of the nerve fiber tracts within peritumoral edema in brain. Neurosurgery 73, 1044–1053. 10.1227/NEU.0000000000000146 24056318

